# Protective Influence of Vaccination

**Published:** 1844-11

**Authors:** 


*On the Protective Influence of Vaccination.*—The general conclusions, drawn by Dr. Retzius of Stockholm from his observations of small pox and the effects of vaccination in Sweeden, are these: "The protection, afforded by vaccination from the close of the second year of life against the contagion of the Variolous poison, usually lasts unimpaired to the end of the thirteenth year or so: after this period it begins to lose its effect, and gradually becomes more and more uncertain on the twentieth or twenty-first year of life. For the next four or five years, the disposition to the small-pox seems almost to have recovered its original integrity; and this state of liability continues unimpaired up to the age of forty years or so. At about this epoch of life, it begins to approach nearer to the limit of its existence—which it reaches, in the majority of cases, about the fiftieth year,—the period when the general revolution of the human body commences to take place."

(The practical inference to be drawn from these remarks is the propriety of repeating vaccination in about thirteen or fourteen years after its first performance. This advice is in accordance with the observations of the most experienced practitioners: it would be well, if it were more generally acted upon.—*Med. Chir. Review.*

